# Diagnosis of Parkinson’s Disease by A Metabolomics-Based Laboratory-Developed Test (LDT)

**DOI:** 10.3390/diagnostics10050332

**Published:** 2020-05-21

**Authors:** Petr G. Lokhov, Oxana P. Trifonova, Dmitry L. Maslov, Steven Lichtenberg, Elena E. Balashova

**Affiliations:** 1 Institute of Biomedical Chemistry, 10 building 8, Pogodinskaya Street, 119121 Moscow, Russia; oxana.trifonova@gmail.com (O.P.T.); dlmaslov@mail.ru (D.L.M.); balashlen@mail.com (E.E.B.); 2 Metabometrics, Inc., 651 N Broad St., Suite 205 #1370, Middletown, DE 19709, USA; sl@biobohemia.com

**Keywords:** metabolomics, Parkinson’s disease, diagnostics, laboratory-developed test, mass spectrometry, blood plasma, biologic context, metabolite identification, putatively annotated metabolites, pathway overrepresentation analysis

## Abstract

A laboratory-developed test (LDT) is a type of in vitro diagnostic test that is designed, manufactured and used in the same laboratory (i.e., an in-house test). In this study, a metabolomics-based LDT was developed. This test involves a blood plasma preparation, direct-infusion mass spectrometry analysis with a high-resolution mass spectrometer, alignment and normalization of mass peaks using original algorithms, metabolite annotation by a biochemical context-driven algorithm, detection of overrepresented metabolic pathways and results in a visualization in the form of a pathway names cloud. The LDT was applied to detect early stage Parkinson’s disease (PD)—the diagnosis of which currently requires great effort due to the lack of available laboratory tests. In a case–control study (*n* = 56), the LDT revealed a statistically sound pattern in the PD-relevant pathways. Usage of the LDT for individuals confirmed its ability to reveal this pattern and thus diagnose PD at the early-stage (1–2.5 stages, according to Hoehn and Yahr scale). The detection of this pattern by LDT could diagnose PD with a specificity of 64%, sensitivity of 86% and an accuracy of 75%. Thus, this LDT can be used for further widespread testing.

## 1. Introduction

Parkinson’s disease (PD) is the second most common neurodegenerative disease of the central nervous system and primarily affects the elderly. In recent years, the incidence of PD has increased dramatically due to the aging population. Today, there are several hypotheses for the pathogenesis of PD, including inflammation, improper protein folding, oxidative stress and mitochondrial damage [1,2]. Researchers tend to view PD as a multifactorial disease and that multiple mechanisms may contribute to its pathogenesis. Due to the multifactorial nature of PD, the discovery of biomarkers for its diagnosis is very complicated and has not yet been successful; therefore, further efforts are required to identify them [3]. The technologies used in the ‘omics’ sciences, which allow measuring the entire diversity of the molecules of a biologic system in a single-run analysis, may help in this situation (e.g., DNA sequencing in genomics, protein identification technologies in proteomics and profiling of low-molecular-weight substances in metabolomics). Currently, metabolomics has become a promising tool for providing valuable information for disease diagnostics because metabolites directly reflect the physiological and pathological conditions in humans. In particular, metabolomics-based studies of blood samples have led to the successful diagnosis of many diseases, with a diagnostic accuracy of 90–95% [4]. Such technological performance gives hope for the implementation of these technologies in medicine. Therefore, researchers are trying to create omics-based tests for diagnosing diseases, assessing the risk of their development and determining the patient’s response to treatment [5].

However, despite the prospect of introducing omics-based tests into the clinic, their application in clinical practice is extremely difficult—mainly because of the lack of regulated procedures and standards [6], the development of which is challenged by their complexity. Therefore, metabolomics assays as ‘laboratory-developed tests’ (LDTs) seem to be a possible solution to this problem.

A LDT is a type of in vitro diagnostic test that is designed, manufactured and used within a single laboratory [7,8,9,10]. LDTs can be used to measure a wide variety of analytes (substances such as proteins, chemical compounds like glucose or cholesterol or DNA) in a sample taken from humans. Some LDTs are relatively simple tests that measure single analytes; while other LDTs, like omics tests, are complex and may measure a large number of analytes. Recently, several LDTs have been published for the diagnosis of various diseases, including genetic disorders, cancers, infections and different other pathological conditions [11,12,13,14,15,16,17]. In this work, the LDT comprising the latest advancements in metabolomics research was tested as an early diagnostic method for PD, which is difficult for diagnosis using available laboratory tests.

The applied LDT workflow included blood sample preparation, high-resolution direct mass spectrometry, an original mass spectrometry peak alignment algorithm, an original mass peak intensity standardization algorithm, a compound annotation algorithm and a statistical model testing the metabolic data of individuals against 808 human metabolic pathways, with output visualization as a pathway names cloud.

## 2. Materials and Methods

### 2.1. Mass Spectra of Blood Plasma

Samples of blood plasma used in this study were taken from a previously published study, where study participants were recruited at the Republican Clinical Diagnostic Centre of Extrapyramidal Pathology and Botulinum Therapy (Kazan, Russia) [18]. Briefly, subjects included untreated PD patients at Hoehn and Yahr Stages 1–2.5 and controls without neurodegenerative disease. The metabolomics study of blood samples was approved by the relevant ethical review committee (the approval number is provided in the original study [18]). All procedures performed in studies involving human participants were in accordance with the ethical standards of the institutional or national research committee and with the 1964 Helsinki Declaration and its later amendments or comparable ethical standards. Table 1 presents the clinical characteristics of the cohort.

Blood samples for metabolomic analysis were taken from the vein before the morning meal. Samples (3 mL) were placed into glass tubes containing K_2_EDTA (BD Vacutainer; Becton, Dickinson and Company, Franklin Lakes, NJ, USA) and centrifuged within 15 min at 1600× *g* and room temperature. The resultant blood plasma was subdivided into aliquots that were pipetted into plastic tubes. These tubes were marked, transported in special thermo containers, frozen, then stored at −80 °C until analysis. The analyzed samples were subjected to one freeze/thaw cycle. For plasma deproteinization, aliquots (10 μL) were mixed with 10 μL of water (LiChrosolv; Merck KGaA, Darmstadt, Germany) and 80 μL of methanol (Fluka, Munich, Germany) and incubated at room temperature. After 15 min, the samples were centrifuged at 13,000× *g* (MiniSpin plus centrifuge; Eppendorf AG, Hamburg, Germany) for 10 min. Deproteinized supernatants were then transferred to clean plastic Eppendorf tubes and fifty volumes of methanol containing 0.1% formic acid (Fluka) were added to each tube. The resulting solutions were subjected to mass spectrometry analysis.

Samples were analyzed with a maXis hybrid quadrupole time-of-flight mass spectrometer (Bruker Daltonics, Billerica, MA, USA) equipped with an electrospray ionization (ESI) source (mass spectra and mass lists are presented in Additional files). The mass spectrometer was set up to prioritize the detection of ions with a mass-to-charge ratio (*m*/*z*) ranging from 50 to 1000, with a mass accuracy of 1–2 parts per million (ppm). Spectra were recorded in the positive ion charge detection mode. Samples were injected into the ESI source using a glass syringe (Hamilton Bonaduz AG, Bonaduz, Switzerland) connected to a syringe injection pump (KD Scientific, Holliston, MA, USA). The flow rate of samples to the ionization source was 180 μL/h and samples were injected in a randomized order (e.g., control samples were run between case samples).

Mass spectra were obtained using DataAnalysis version 3.4 (Bruker Daltonics) to summarize one minute signals. Ion metabolite masses were determined from the mass spectrum peaks obtained using the DataAnalysis program. All peaks above noise level (signal to noise ratio >1) were selected, and the metabolite ion masses were pooled and further processed.

### 2.2. Mass Spectra Preprocessing

Recalibration, peak detection and peak intensity calculation of mass spectra were carried out automatically by DataAnalysis software. Masses of compounds were determined from the mass spectrum peaks obtained using the following parameters: peak width, 2; signal-to-noise ratio, 1; relative and absolute threshold intensity, 0.01% and 100, respectively. For recalibration of all the peak intensity values, the internal standard losartan (*m*/*z* 423.169) was used.

Normalization of mass peak intensities was performed as described previously [20] by the data preprocessing block of the in-house software. The alignment of the *m*/*z* values of the mass peaks to the different mass spectra was performed as described previously [21] by the same block of the in-house software. The resulting *m*/*z* values with a nonzero mass peak intensity for more than nine samples were submitted to the metabolite search engine block of the in-house software.

### 2.3. Search for Correspondence of Mass Peaks to Metabolite Identifiers

The search for correspondence of each mass peak to metabolite identifiers was done by the database search engine block of the in-house software. The human metabolome database (HMDB; www.hmdb.ca) was used as the source of the *m*/*z* values and KEGG IDs. For this, the database in the XML format was downloaded and parsed, and all metabolites with KEGG IDs were collected with molecular weights. The search engine determined the correspondence between the *m*/*z* values and the KEGG identifiers, with a maximum error of 0.005 Da and taking into account the positive mode of ionization (corrected for H^+^, Na^+^ and K^+^ adducts) and the presence isotopes in the mass spectra. The retrieved list of metabolite names for each *m*/*z* value was further processed by the compound annotation block of the in-house software.

### 2.4. Compound Annotation Algorithm

A compound annotation algorithm was recently developed and described in detail [22]. This algorithm uses metabolic pathway data and allows for the effective annotation of low-molecular-weight blood components (metabolome) with relatively high speed. In the list of compound names, many candidates, on average, were associated with one mass. The main task of the algorithm is to compare the obtained experimental data, i.e., mass spectra, with the available information on biochemical pathways and to decline all false candidates. It is known that the concentrations of compounds involved in the same pathways correlate [23]. Thus, if mass spectrometry data for a set of samples are available, the correlation between the mass of interest and other mass peaks can be found. The masses of these correlating peaks can also be associated with a set of compounds whose locations in the metabolic pathway must be bunched around the compound with the true annotation. The details of the application of this algorithm for blood plasma samples have been described previously [22]. The next release of this algorithm with an updated source code to make it more suitable for blood samples was used in the LDT.

### 2.5. Pathway Overrepresentation Analysis

Analyzed HMDB data were used to compile a matrix in which the rows correspond to 808 metabolic pathways and the cells in the matrix contain the identifiers of the metabolites associated with these pathways. Selected metabolites in the case–control or individual sample studies were projected on these pathways, and the obtained results were compared with the projections, which were performed 30,000 times with the same number of randomly selected metabolites. The obtained results were normalized to produce a pathway representation score for each pathway as the LDT output reflecting the pathway representation. This score also was used to visualize the results as a pathway names cloud in which the font size is related, although not directly, with the score. This allowed the visualization of the overrepresented pathways. The scores for the top 20 overrepresented pathways were summarized to produce the final diagnostic score for each person who participated in the study. Overrepresentation analysis was performed by the corresponding block of the in-house software.

### 2.6. Detection of the PD Pattern by the LDT

To reveal the PD-associated pattern in metabolic pathways, the mean scores for cases were compared with those of the controls. Additionally, the scores were analyzed by the Wilcoxon rank-sum test (by an imbedded function of the in-house software).

### 2.7. Analysis of Individual Samples by the LDT

To reveal metabolites associated with up- or downregulated pathways, the Z-score was calculated for mass peak intensities. The mean value and standard deviation included in the Z-score equation were calculated only using control samples. For the control samples themselves, the leave-one-out approach was also used. Metabolites with a Z-score > 1.64 or < −1.64 were considered as metabolites with increased and decreased concentrations, respectively, and were submitted to pathway overrepresentation analysis.

**Figure 1:**
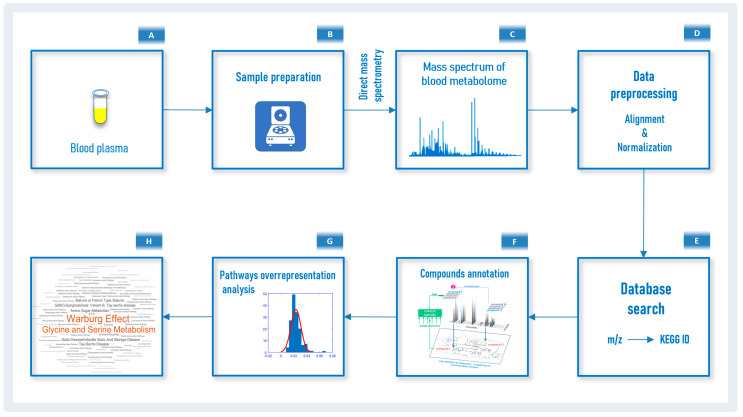
Laboratory-developed test workflow. Blood plasma samples (**A**) are collected and transported to the laboratory. In the laboratory, after sample preparation (**B**) and using high-resolution mass spectrometry, the mass spectra of blood plasma samples are obtained (**C**). The obtained mass spectra after preprocessing (**D**) are submitted to the database search engine (**E**) to find metabolite identifier from Kyoto Encyclopedia of Genes and Genomes (KEGG IDs) matching the mass-to-charge ratio (*m*/*z*) values. A list of matched KEGG IDs is analyzed according to a compound annotation algorithm (**F**) [22] and the retrieved results are used for the overrepresented pathways analysis (**G**). Finally, overrepresented pathway results from an individual are visualized as a pathway names cloud, where the font size corresponds with the representation value (**H**).

The workflow used in this study to analyze blood plasma samples by the LDT from patients with PD is presented in Figure 1. The proprietary in-house software, which is the bioinformatic part of the LDT, was consistently used for data preprocessing, database searching and overrepresentation analysis, and the output was implemented in Matlab. To perform all calculations, a Sony-VAIO (Intel^®^ Core™ i7-2640 CPU 2.80 GHz, Windows 10 Pro) personal computer was used.

## 3. Results

### 3.1. Mass Spectrometry Analysis of Compounds in Blood

Mass spectrometry analysis, as the first analytical block of the LDT workflow (Figure 1), generated typical mass spectra of the low-molecular-weight fraction of blood plasma samples. On average, 9664 peaks were detected in the spectrum (Table 2). The mass peaks of compounds were submitted to the metabolite search engine block of the LDT to annotate the compounds matching the mass peaks; 31,724 records of compound names with an associated molecular weight corresponding to the measured value of mass-to-charge ratio (*m*/*z*) were retrieved.

### 3.2. Compound Annotations

From the 31,724 records of compound names associated with *m*/*z* values, the annotation algorithm selected 2741 candidates as putatively true. The application of the annotation algorithm resulted in the annotation of 709 compounds (Table 2). Among the annotated compounds, amino acids, steroid hormones, fatty acids and other lipids, nucleotides, carbohydrates and different compound derivatives were mainly presented.

### 3.3. Pathway Pattern of PD

A case–control study revealed a detectable pattern of PD by the LDT. The pathway representation scores for the control samples were compared with the scores for the samples from PD patients. The top 20 overrepresented metabolic pathways for the PD patients are listed in Table 3.

**Figure 2:**
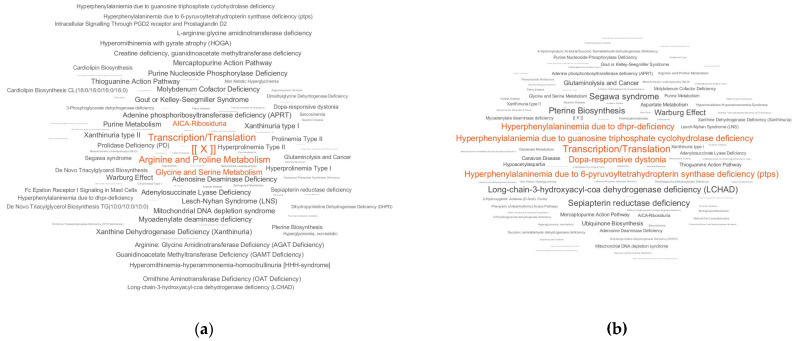
Laboratory-developed test (LDT) output for the ‘case–control’ study of Parkinson’s disease (PD). (**a**) LDT output for controls, showing in what pathways metabolites are detected by the test; (**b**) LDT output for cases. The marker [ [X] ] has the same value on both plots and is provided for plot comparison. The pathways with the top five scores are colored in red. The LDT reveals that the pattern of early stage PD includes overrepresentation in transcription/translation and nervous system-related pathways (dopa-responsible dystonia, hyperphenylalaninemia, pterine biosynthesis, Warburg effect, Segawa syndrome, etc.).

The LDT generated the output as a pathway names cloud for the controls and cases (Figure 2). Thus, a PD-associated pathway pattern was found in metabolic data of blood plasma.

### 3.4. Diagnosis of PD by LDT

**Figure 3:**
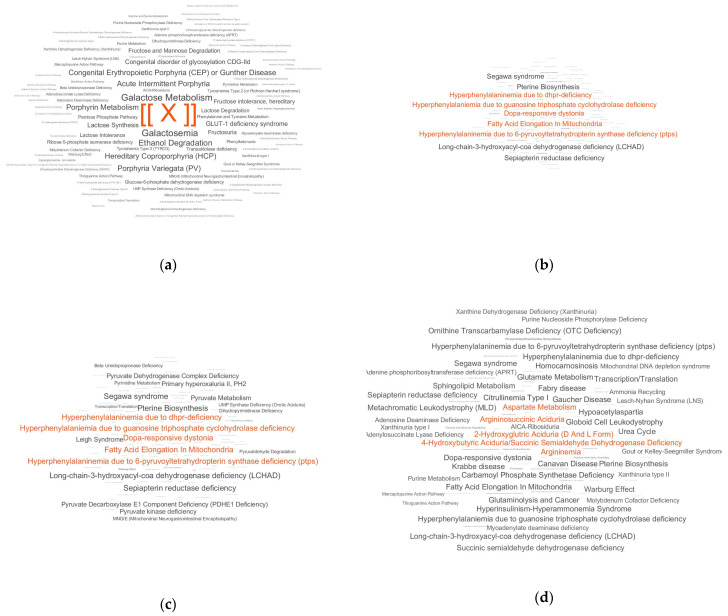
Laboratory-developed test outputs for four individuals. Typical outputs for a control patient (**a**), a patient with Parkinson’s disease (PD) and a detectable PD-associated pathway overrepresentation pattern (**b**), another person with PD with the same pattern (**c**) and a PD patient with a PD pattern accompanied with overrepresentation of other metabolic pathways (**d**). The marker [ [X] ] has the same value on all plots and is provided for plot comparison. The overrepresented pathways with the top five scores are colored in red.

For the volunteers from the control group, the LDT showed the absence of metabolic disorders as well as various personal deviations for particular patients, possibly associated with the presence of certain non-neurodegenerative diseases. The PD-associated pattern was detected in four controls (the most expressed pattern was associated with the oldest volunteer (71 years) in the control group). A typical LDT output for a control subject without any overrepresentations is presented in Figure 3a. Figure 3b demonstrates a detectable PD pattern for a PD patient. To show how the PD pattern can look different for another sick person, Figure 3c is provided. Figure 3d demonstrates the same pattern accompanied by other overrepresented metabolic pathways in a PD patient with additional health concerns.

**Figure 4:**
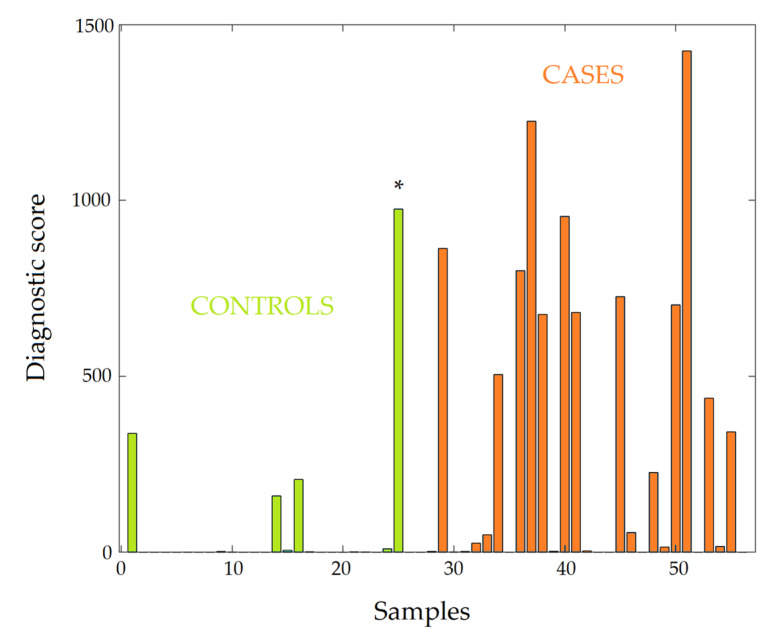
Diagnostic scores of laboratory-developed test for control subjects (green bars) and patients (red bars) with Parkinson’s disease (PD). The diagnostic score is produced by summarizing the pathway representation scores for the top 20 overrepresented PD pathways (see Table 3). * indicates the oldest subject (71 years old) in the control cohort.

The clear identification of the PD pattern in most patients made it possible to suggest the diagnosis of PD based on the LDT. Figure 4 shows the LDT diagnostic scores (sum of 20 pathway representation scores) for cases and controls, and Table 4 shows the diagnostic criteria for diagnosing early stage PD. The accuracy of diagnosis was 75%, thus demonstrating that the LDT is an efficient diagnostic tool for the early diagnosis of PD. Given the existing diversity in the pathogenesis of PD, an additional threshold value of the score, separating cases and controls, was chosen. It provides more reliable detection of the identified PD pattern and corresponds to a high diagnostic score. If this pattern is detected, it is almost 100% certain that the patient has PD.

## 4. Discussion

PD is a progressive degenerative disease of the central nervous system that is mainly diagnosed based on the medical history and neurological examination [24]. The early diagnosis of PD is necessary for effective therapy [25], but a clinical diagnosis of PD is not possible until a significant loss of dopaminergic neurons occurs [26]. The imaging of dopamine (Dopa) uptake efficiency as a diagnostic test is expensive and has a limited availability. Therefore, a diagnostic laboratory test, which is currently unavailable, is urgently needed. However, the uncertain pathogenesis and multifactorial nature of PD have hindered biomarker discovery and the use ‘panoramic’ approaches has been suggested as a possible tool for PD diagnostics. Unfortunately, the clinical application of such ‘panoramic’ methods, to which metabolomic profiling is related, is extremely complicated due to the standardization required for clinical test registration. The usage of LDTs overcomes this obstacle. The Food and Drug Administration (USA) considers LDTs as tests that are designed, manufactured and used inside the same laboratory [7]. It sufficiently simplifies the implementation of metabolomics testing, thus bringing protocol development and standardization activities to single laboratory routines.

The developed LDT is based on direct mass spectrometry of blood plasma. It has been successfully used to study cancer [21,27,28,29,30], diabetes [31] and even PD [18]. Direct mass spectrometry is characterized by a high processing speed and a high reproducibility of data [32,33,34], which are useful for clinical purposes. The mass spectrometry peaks alignment algorithm was specially designed for high-resolution mass spectra [21]; an updated version of this algorithm is implemented in the LDT. The data standardization algorithm used in the LDT was previously developed for high-resolution mass spectra of blood metabolites to simplify their use in clinical metabolomics [20].

The creation of an LDT block for identifying metabolites based on mass spectrometry data required special efforts, as this is the bottleneck of metabolomics. As a general rule, the mass spectrometry methods used in metabolomics studies can detect hundreds of compounds, which is crucial for obtaining biochemical information [35]. Unfortunately, even if the most advanced metabolomics technologies using mass spectrometry are used, the vast majority of compounds in the sample remain unknown [36]. Usually, only well-separated and abundant metabolites are identified. The reason for this is that a clear mass spectrometric picture of a substance or its fragments is necessary to compare it with database data and obtaining such a picture for low-abundance metabolites, which make up most metabolites in any mass spectra, is a big problem. This has led to the emergence of new approaches for identifying compounds. The biochemical context-driven annotation (i.e., identification with a low probability) of compounds is one such approach that takes into account knowledge of their biotransformation in biochemical pathways. An excellent example of this methodology was developed by Rogers and coworkers [37] and further advanced by Silva and coworkers [38]. The first application of this approach for the putative annotation of metabolites in complex samples, like blood plasma, was applied in our laboratory [22]. This updated approach for complex samples was implemented in the LDT, which allowed the annotation of more than 700 metabolites, on average, per sample. According to the Metabolomics Standards Initiative standard [39], metabolite annotations obtained by the LDT are related to level 2 of metabolite identification (‘putatively annotated compounds’) because two independent orthogonal features of each metabolite are used for annotation (accurate mass tag and biochemical context). Further representation assessment of pathways with a certain number of annotated metabolites provides scores that reflect the detected overrepresentation in a particular pathway.

As the first step in the testing of the LDT, a ‘case–control’ approach was used. The LDT detected numerous PD-associated overrepresented pathways. Notably, almost all of the overrepresented pathways (Table 3) are relevant to PD. For example, deregulated transcription and translation are currently described in PD [40,41]. In addition, it is a prominent fact that Dopa is related to PD [42]. Dysregulated lipid metabolism and the role of mitochondria are also described for PD [43]. Moreover, the pterin synthesis pathway is directly connected with monoamine neurotransmitters, and pterin metabolites can be used for the diagnosis of Dopa-responsive dystonia. Segawa syndrome is the same as autosomal dominant Dopa-responsive dystonia and guanosine triphosphate cyclohydrolase deficiency. Furthermore, 6-pyruvoyltetrahydropterin synthase deficiency is a neurodegenerative disease like PD that is treated by levodopa; similarly, dihydropteridine reductase (DHPR) deficiency; the inverse Warburg effect is associated with neurodegenerative diseases like Alzheimer’s disease and PD. Altogether, these data provide additional evidence that the LDT-detected pattern is based on relevant data. Notably, the PD pathway was not presented in the LDT output because there is no such pathway.

The next step of this study was the application of the LDT for samples from individuals. In this experiment, there was no chance to extract case-associated features like in the ‘case–control’ approach. Nonetheless, the LDT detected a PD pattern in pathways for most case samples. In some situations, this pattern was detected clearly (Figure 3b,c). In other situations, the pattern was accompanied by the overrepresentation of other pathways (Figure 3d) because relatively old subjects participated in the study and other diseases are present in their bodies. It should be noted that the LDT is not tuned to detect only PD. It is a metabolomics-based assay that covers a variety of diseases, i.e., the PD pattern in the LDT output may be presented among patterns of other diseases. To simplify PD diagnostics for such situations, the diagnostic score calculation is implemented in the LDT. The score-based diagnostics provided 75% accuracy, thus showing that this LDT is a very promising tool for PD diagnostics at the early stage.

## 5. Conclusions

The metabolomics-based LDT was developed; and its ability to diagnose PD, a disease that is extremely difficult to diagnose by laboratory tests, was confirmed. The success of this early stage PD diagnostic method and the omics nature of the LDT suggest that it can be used for a variety of diseases; therefore, further widespread assessment of this LDT should be implemented. Moreover, the compatibility of the high-resolution mass spectrometry of blood metabolites with a dried blood spot (DBS) [44] eliminates the main drawback inherent in all LDTs—its localization in a specific laboratory—because a DBS can be obtained both in the clinic and without assistance at home, followed by transportation on a study card at room temperature, thus making the LDT available everywhere there is mail service.

## Figures and Tables

**Table 1:** Study cohort characteristics.

Characteristics	Values	
	Control Subjects	Subjects with PD ^1^
Number	28	28
Age (years; mean ± *s.d.* (range)	62.8 ± 8.7 (45–77)	62.6 ± 8.6 (37–77)
Gender (male/female)	14/14	14/14
PD stages (1/1.5/2/2.5)	–	6/6/12/4

^1^ PD stages are according to Hoehn and Yahr scale [19].

**Table 2:** Variables associated with this study.

Parameter	Value
Detection mass range of compounds (*m*/*z*)	45–900
Number of detected compound mass peaks	9664 ± 620 ^1^
Number of masses submitted to search engine block	14,857
Number of mass peaks/compound candidate submitted to the annotation algorithm	31,724
Number of mass peaks with putatively annotated compound(s) by the annotation algorithm	2741
Number of unique compound names retrieved by the annotation algorithm	709

^1^ average ± standard deviation.

**Table 3:** Summary of laboratory-developed test (LDT) outputs for control and case subjects.

Pathway	Pathway Representation Score ^1^		Pathway Overrepresentation (Fold)	Wilcoxon Rank-Sum Test
	Case Samples	Control Samples		
Transcription/translation	27.8	8.6	3.2	0.003
Dopa-responsive dystonia	21.4	2.7	7.9	0.3
Fatty acid elongation in mitochondria	21.4	2.7	7.9	0.3
Long-chain-3-hydroxyacyl-coa dehydrogenase deficiency (LCHAD)	21.4	2.7	7.9	0.3
Hyperphenylalaninemia due to guanosine triphosphate cyclohydrolase deficiency	21.4	2.7	7.9	0.3
Hyperphenylalaninemia due to 6-pyruvoyltetrahydropterin synthase (PTPS) deficiency	21.4	2.7	7.9	0.3
Hyperphenylalaninemia due to DHPR deficiency	21.4	2.7	7.9	0.3
Pterine biosynthesis	21.4	2.7	7.9	0.3
Segawa syndrome	21.4	2.7	7.9	0.3
Sepiapterin reductase deficiency	21.4	2.7	7.9	0.3
Warburg effect	20.3	4.6	4.4	0.0004
Glutaminolysis and cancer	14.4	2.9	5.0	0.029
Mercaptopurine action pathway	11.7	4.6	2.5	0.24
Thioguanine action pathway	11.7	4.6	2.5	0.014
Glycine and serine metabolism	9.4	5.3	1.8	0.014
AICA-ribosiduria	9.4	4.7	2.0	0.014
Adenine phosphoribosyltransferase deficiency (APRT)	9.4	4.7	2.0	0.36
Adenosine deaminase deficiency	9.4	4.7	2.0	0.36
Lesch–Nyhan Syndrome (LNS)	9.4	4.7	2.0	0.004
Mitochondrial DNA depletion syndrome	9.4	4.7	2.0	0.29

^1^ mean value.

**Table 4:** Criteria for LDT diagnostics for PD.

Criteria	Value	
	Score Threshold #1	Score Threshold #2
		
Score threshold	12	340
		
True positive	18	12
False positive	4	1 ^1^
True negative	24	27
False negative	10	16
		
Sensitivity	64%	43%
Specificity	86%	96%
Accuracy	75%	70%

^1^ corresponds to the oldest subject (71 years old) in the control cohort.
